# Refining the Intergenerational Technology Checklist for Higher Education through a Delphi Panel

**DOI:** 10.1093/geroni/igaf122.3442

**Published:** 2025-12-31

**Authors:** Jill Juris, Skye Leedahl, Shannon Jarrott, Caroline Halliday

**Affiliations:** Appalachian State University, Boone, North Carolina, United States; University of Rhode Island, Kingston, Rhode Island, United States; The Ohio State University, Columbus, Ohio, United States; Appalachian State University, Boone, North Carolina, United States

## Abstract

The aim of this study was to evaluate the InterGenerational Technology Engagement Checklist for Higher Education (IG TECH) using the Delphi approach that builds expert consensus. We conducted three rounds with the Delphi panel comprised of researchers and practitioners with expertise in intergenerational programs. In each round, participants evaluated the IG TECH using a Criteria Rating Form that included quantitative and qualitative responses related to: 1) Construction, 2) Appropriateness and, 3) Relevance. Responses on a four-point Likert scale ranged from “Poor” to “Excellent”. Consensus was pre-defined as 80% of ratings for each item “Good” or “Excellent.” The Criteria Rating Form was modified in subsequent rounds to include items with less than 80% approval. Participants were invited from a repository of 283 programs identified through the Generations United database, previous research participation, and gerontology networks. Of those, 47 participants responded to the interest survey, and 39 responded to the Round 1 survey (83% response rate). In Round 2, the Round 1 panelists were invited, and 33 completed the questionnaire (85% response rate). In Round 3, the final round, Round 2 participants were invited and 30 responded (91% response rate). In each round, the research team reviewed quantitative and qualitative data from the Criteria Rating Form and identified specific changes to the IG TECH. Changes were categorized and shared with the Delphi panel. In Round 3, panelists reached 100% consensus on all items. A final version of the IG TECH was developed that includes six sections and a total of 45 items.

